# Institutional Questions

**Published:** 1900-09-29

**Authors:** 


### INSTITUTIONAL QUESTIONS.

### Disinfection.

"Architect" asks: Is a germ destructor an absolutely necessary adjunct to a fever hospital, or is there any other way of dealing with infected clothing and bedding less costly?

⁂ The only known method of obtaining a satisfactory disinfection is by steam. It is as easy to get a complete disinfection by low-pressure steam as by what is called superheated steam. The one important thing is that the steam shall blow right through the disinfector what is called "current steam," and the best direction is that it shall blow off at the bottom. It must go right through. The thing that costs the money in a disinfector is having the steam at a pressure which, to be safe, involves the whole apparatus being very strong, and therefore very expensive. There is this great advantage in a superheated steam arrangement, that the clothes come out dryer than from a saturated steam arrangement, and for daily use this is worth consideration. For a small hospital, which half its time may be standing empty, it may not be worth while to go to the expense involved in using high-pressure steam, and in employing the skilled attention that high-pressure steam always requires. There is no machinery in a disinfector. If one has a supply of steam, it is perfectly possible to make a completely efficient disinfector out of sheet tin. Directly you have pressure, however, you must have safety valves, and all the rest of it.

### Cubic Space.

"Enquirer" asks: Could the size of a two-bed ward in a fever hospital be reduced to less than 2,000 feet of air space per bed if the hospital is situated in a salubrious climate where the windows can be open nearly every day throughout the year?

⁂ We cannot recommend that the air space should be lessened. It is really more important that there should be a good cubic space in a small fever hospital than in a large one, because in a small one it is not always possible to ensure the same completeness of classification that one can get where more beds are at one's disposal. It might, for example, be necessary to treat two different diseases in the same ward, so that a full cubic space becomes very necessary. We quite see that a climate which allows the windows to be open is an advantage; but unless there is a breeze it is really in the warm weather that the difficulties of ventilation are often the greatest, and one must never forget that a large cubic space gives us not merely more air, but the power of placing the beds further apart.

